# Quality of life at work

**DOI:** 10.47626/1679-4435-2025-232

**Published:** 2025-06-26

**Authors:** Paula Cristina Moreira Couras da Silva

**Affiliations:** Associate Editor, Revista Brasileira de Medicina do Trabalho

It is with great pleasure that we present the latest issue of the Revista Brasileira de Medicina do Trabalho, reaffirming our commitment to sharing high-quality, ethical technical-scientific knowledge with readers in the field of occupational medicine and workers’ health.

We would also like to highlight the considerable interest generated by our previous issue, which focused on Psychosocial Factors at Work. The published articles received an average of 1200 views by the end of May, which demonstrates the national relevance and timeliness of the topic. This growing interest reflects the evolving world of work, the challenges organizations face in promoting workers’ mental health, and the importance of a broader view of psychosocial working conditions.

Despite the recent postponement of inspection by the Brazilian Ministry of Labor and Employment concerning the inclusion of psychosocial risk factors in Occupational Risk Management, it is important to emphasize that psychosocial aspects are an integral part of several Regulatory Standards (Normas Regulamentadoras [NRs]), such as 05 (Internal Accident Prevention Committee), 07 (Occupational Health Medical Control Program), 17 (Ergonomics), 20 (Flammable and Combustible Materials), 33 (Confined Spaces), 35 (Work at Height), 36 (Meatpacking Plants), and 37 (Oil Rigs), all of which feature specific provisions for assessing psychosocial factors at work.

These NRs recognize psychosocial risks as relevant factors resulting from deficiencies in work design, organization, and management, which can negatively impact the mental, physical, and social health of workers. Furthermore, eSocial, the digital recording platform for labor and social security obligations, includes in its table 23 ergonomic/organizational items (codes 04.03.001 to 04.03.010) and psychosocial/cognitive items (codes 04.05.001 to 04.05.010), a comprehensive list of psychosocial risk factors at work and in the organization, underscoring the importance of the topic for the integrated management of health and safety at work.

In this context, occupational physicians and other occupational safety and health professionals play a key role in this process. It is essential that they are attentive to the careful assessment of psychosocial factors at work and the appropriate management of these factors, recognizing them as opportunities for workers’ overall health promotion and prevention, acting in a technical, ethical, and interdisciplinary manner.

We hope you enjoy reading this issue and that it contributes to the continuous improvement of occupational health practices in Brazil.

Paula Cristina Moreira Couras da Silvahttps://orcid.org/0000-0002-5647-3590

Associate Editor, Revista Brasileira de Medicina do Trabalho

